# Clinical-Pathological Conference Series from the Medical University of Graz: Case No 125: A 42-year-old man with loss of appetite, vomiting and stabbing abdominal pain

**DOI:** 10.1007/s00508-022-02025-y

**Published:** 2022-05-11

**Authors:** Elisabeth Fabian, Vanessa Stadlbauer, Felix Keil, Karin Hegenbarth, Eckhard Beubler, Guenter J. Krejs

**Affiliations:** 1 Division of Gastroenterology and Hepatology, Department of Internal Medicine III, Medical University of Vienna, Vienna, Austria; 2 Division of Gastroenterology and Hepatology, Department of Internal Medicine, Medical University of Graz, Auenbruggerplatz 15, 8036 Graz, Austria; 3 Department of Internal Medicine 3, Hematology and Oncology, Hanusch Hospital, Vienna, Austria; 4 Poliklinik Rüdersdorf bei Berlin, Rüdersdorf, Germany; 5 Department of Clinical Pharmacology and Toxicology, Medical University of Graz, Graz, Austria

**Keywords:** Lead poisoning, Pseudoacute abdomen, Anemia, Lead line, Chelating agents

## Presentation of case

### Dr. V. Stadlbauer:

The patient had spent several days working on the demolition of an old railway bridge 6 weeks prior to admission. He used a cut-off wheel and welding apparatus for the demolition, generating considerable dust. In the following days, the patient developed bursitis of the right knee; he was treated with cefpodoxime for 5 days but was not given a non-steroidal anti-inflammatory drug (NSAID). A few days after finishing his course of antibiotics, the patient lost his appetite and vomited repeatedly. He also complained of constipation and had not moved his bowels for 3 days prior to admission. He had persistent stabbing pain in the mid and lower abdomen that was independent of eating. On admission, the patient reported having lost 12 kg over the previous 6 weeks. A few days before admission to the Department of Internal Medicine at Graz University Medical Center he had been examined in a peripheral hospital. Both the esophagogastroduodenoscopy and colonoscopy were unremarkable and no cause for his complaints was found. The patient worked as a mechanic for the Austrian Federal Railway System and had regular medical check-ups that were always unremarkable. He reported to be a nonsmoker and social drinker. The patient took no medication besides that mentioned above. His past medical history included an appendectomy, tonsillectomy and thoracic surgery after a knifing incident. He had not travelled abroad.

Physical examination revealed an afebrile patient in good general health with a body height of 178 cm and a body weight of 78 kg (body mass index, BMI, of 25 kg/m^2^). His sclerae were slightly icteric. Further clinical examination revealed rhythmic heart sounds and no murmurs, and vesicular breath sounds in both lungs. The abdominal wall was soft, there was diffuse tenderness on palpation in the entire lower abdomen; bowel sounds were unremarkable. The spleen could not be palpated, the extremities and cursory neurological examination were normal.

Laboratory data: Serum creatine kinase 36 U/L (normal: < 170 U/L), lactate dehydrogenase 201 U/L (normal: 120–240 U/L), aspartate amino transferase (AST) 50 U/L (normal: < 35 U/L), alanine amino transferase (ALT) 104 U/L (normal: < 45 U/L), gamma-glutamyl transferase (GGT) 88 U/L (normal: < 15 U/L), alkaline phosphatase 83 U/L (normal: 55–70 U/L), total bilirubin 1.9 mg/dL (normal: 0.1–1.2 mg/dL), creatinine 1.3 mg/dL (normal: 0.6–1.3 mg/dL), urea 40 mg/dL (normal: 10–45 mg/dL), glucose 114 mg/dL (normal: 70–115 mg/dL), *p*-amylase 33 U/L (normal: < 53 U/L), lipase 40 U/L (normal: < 60 U/L), pseudocholinesterase 8665 U/L (normal: 3900–13,000 U/L), sodium 140 mmol/L (normal: 135–145 mmol/L), potassium 4.6 mmol/L (normal: 3.5–5.0 mmol/L), chloride 100 mmol/L (normal: 95–105 mmol/L), total calcium 2.66 mmol/L (normal: 2.20–2.65 mmol/L), total protein 6.9 mg/dL (normal: 6.6–8.3 mg/dL), C‑reactive protein (CRP) and serum lipids were all normal. Blood count: Leukocytes 6.83 G/L (normal: 4.40–11.30 G/L), erythrocytes 3.78 T/L (normal: 4.50–5.90 T/L), hemoglobin 11.0 g/dL (normal: 13–17.5 g/dL), hematocrit 21% (normal: 40–50%), mean corpuscular volume (MCV) 86 fL (normal: 80–98 fL), mean corpuscular hemoglobin (MCH) 29 pg (normal: 28–33 pg), platelets 264 G/L (normal: 150–450 G/L). Differential blood count: neutrophils 56% (normal: 50–75%), lymphocytes 31% (normal: 20–40%), monocytes 12% (normal: 2–12%), eosinophils 0.9% (normal: < 5%), basophils 0.3% (normal: < 1%), reticulocytes 54 ‰ (normal: 5.0–20.0 ‰). Haptoglobin was 0.56 g/L (normal: 0.3–2.0 g/L), Coombs test was negative. Bone marrow aspirate showed a mildly to moderately hyperplastic bone marrow with slight to moderate hyperplastic left-shifted erythropoiesis, along with a moderately elevated hemosiderin content. Prothrombin time 99% (normal: 70–125%), activated partial thromboplastin time (aPTT) 30 s (normal: 27–41 s), serum iron 218 µg/dL (normal: 50–160 µg/dL), ferritin 553 ng/mL (normal: 34–310 ng/mL), transferrin 1.65 g/L (normal: 2.00–3.60 g/L). Urinalysis was within normal limits. Tests for viral hepatitis (A, B, C), human immunodeficiency virus, adenovirus, *Chlamydia pneumoniae*, human herpesvirus 8, Coxsackie virus, cytomegalovirus, Epstein-Barr virus, herpes simplex virus 6, and leptospirosis were all negative. Screening for antinuclear antibodies (ANA), antibodies against granulocyte cytoplasm (ANCA) and antimitochondrial antibodies (AMA) were negative.

Echocardiogram showed sinus rhythm with a HR of 75 bpm, horizontal heart, unremarkable PQ, and concordant ST segments. Abdominal sonography showed diffuse hyperechogenicity of the liver and an unremarkable gallbladder. The spleen was of normal size and both kidneys were unremarkable. Plain abdominal X‑ray showed the ascending colon and right colonic flexure filled with stool, the transverse colon distended with gas, but no evidence of pathologically dilated intestinal loops, air-fluid levels or free air. Magnetic resonance imaging (MRI) and magnetic resonance cholangiopancreatography (MRCP) showed unremarkable liver, bile ducts and spleen. In the head of the pancreas, there was evidence of an oval hyperintensity measuring 1.2 cm (T2-weighted) near the duodenum. Computed tomography (CT) of the abdomen indicated that the lesion identified on MRI was most likely a small duodenal diverticulum. Besides an area of focal steatosis in the gallbladder bed, the liver was otherwise unremarkable as were the spleen, the adrenal glands and the pelvic organs. The maximum size of the mesenteric lymph nodes was 8 mm; the pelvic and inguinal lymph nodes were not enlarged. The CT further revealed small cysts in the left renal cortex and diverticular disease of the sigmoid colon.

A diagnostic test was performed.

## Differential diagnosis

### Dr. F. Keil:

This is a remarkable case of a patient with normocytic normochromic anemia, reticulocytosis, and elevated serum iron and ferritin. Further, his bilirubin and transaminases were slightly increased. The patient presented clinically with loss of appetite, repeated vomiting, lower abdominal pain, constipation and weight loss. In view of this constellation of findings, one may first think of cancer; however, abdominal sonography, esophagogastroduodenoscopy and colonoscopy were unremarkable and did not find any gastrointestinal cancer or other digestive diseases, such as chronic gastritis, inflammatory bowel disease or pseudomembranous colitis (after taking antibiotics). Since the MRI and CT revealed a duodenal diverticulum with a diameter of 1.2 cm, and laboratory data showed moderate cholestasis, Lemmel’s syndrome should also be included in this patient’s differential diagnosis. Lemmel’s syndrome is an uncommon pathology which was first described in 1934 [1] and is defined as obstructive jaundice due to a periampullary duodenal diverticulum in the absence of choledocholithiasis or neoplasm. The prevalence of duodenal diverticula is around 17% and increases with age [2]. Most are extraluminal and acquired, and predominantly located in the second portion of the duodenum close to the ampulla of Vater (juxtapapillary) where the intestinal wall is weaker. Only 5% are symptomatic [3]; the majority of duodenal diverticula are found incidentally. The existence of a juxtapapillary diverticulum predisposes to biliopancreatic disease due to extrinsic compression of the bile duct by the diverticulum, but also by promoting bacterial overgrowth which facilitates biliary lithiasis due to beta-glucuronidase activity and induces sphincter of Oddi dysfunction, leading to stasis and biliary reflux from the duodenum to the common bile duct [4]. In this case, however, the finding of unremarkable bile ducts on MRCP and the lacking history of epigastric pain excluded the diagnosis of obstructive cholestasis secondary to duodenal diverticulum. Indeed, several imaging studies carried out in this patient (abdominal sonography, abdominal X‑ray, MRI, MRCP and CT) were all unremarkable, making diseases of the stomach, duodenum, gallbladder and pancreas, as well as conditions, such as partial obstruction or volvulus, rather unlikely in this case.

A gastrointestinal disorder characterized by abdominal pain and altered bowel habits (constipation, diarrhea or both) in the absence of structural abnormalities as observed in the discussed patient is the irritable bowel syndrome (IBS). This is thought to be a disorder of the young, with most new patients presenting before the age of 45. Our 42-year-old patient falls into this group. Abdominal pain or discomfort is a prerequisite clinical feature of IBS and is highly variable in intensity and location. Pain is often exacerbated by eating or emotional stress and relieved by passage of flatus or stools. Alteration in bowel habits with episodes of diarrhea and constipation is another typical clinical feature of IBS [5]; however, nocturnal diarrhea as found in diarrhea due to organic causes does not occur in IBS [6]. Inflammation, bleeding, malabsorption and weight loss are not present in IBS [5]. Thus, a weight loss of 12 kg as documented in the discussed patient rules out IBS as a differential diagnosis in this case. Moreover, IBS is hardly ever chosen for discussion in clinical-pathological conferences.

Abdominal pain without the finding of any structural or functional pathology suggests a so-called pseudoacute abdomen mimicking acute abdomen in our patient. As summarized by Bockus in 1958 [7], a variety of diagnoses can cause this condition (Table 1). These include, among others, acute intermittent porphyria, diabetic pseudoperitonitis, sickle cell crisis, chronic lead poisoning and some infectious diseases such as leptospirosis, malaria, hantavirus infection, typhoid fever or herpes zoster. Since the patient was afebrile, inflammatory parameters were not increased, serology for different infections including herpes zoster and leptospirosis were negative, and the patient had not travelled abroad (ruling out malaria as a differential diagnosis), an infection seems unlikely to be the underlying cause for his condition.

**Table 1:** Causes of pseudoacute abdomen [7–9]

Acute intermittent porphyria
Diabetic pseudoperitonitis
Sickle cell crisis
Chronic lead poisoning
Addison’s disease
Familial Mediterranean fever
Spontaneous bacterial peritonitis
Infectious diseases (typhoid fever, hantavirus, malaria, leptospirosis)
Vasculitis (Henoch-Schönlein purpura, periarteritis nodosa)
Hereditary angioedema
Acute glaucoma
Proptosis
Abdominal epilepsy
Myocardial infarction, acute pericarditis
Dissection of the abdominal aorta
Basal pneumonia, lung infarction, pleurodynia
Hematoma of the abdominal wall (hematoma of the rectus muscle due to anticoagulation)
Herpes zoster

Given that the patient presented with normocytic normochromic anemia with increased reticulocytes (which is the physiologic response to anemia), metabolic-toxic causes, porphyria and diseases causing hemolysis are top candidates as differential diagnosis in this case. Hemolytic anemia can be classified according to whether (1) the abnormality is intrinsic or extrinsic to the red blood cell (intracorpuscular versus extracorpuscular defects), (2) the condition is inherited or acquired, (3) the hemolysis is acute or chronic, (4) the mechanism involves antibody-mediated destruction (immune versus nonimmune mechanism) and (5) the hemolysis occurs in the vasculature or in the reticuloendothelial system in the liver and spleen (intravascular versus extravascular hemolysis) ([10]; Table 2). The normal level of haptoglobin does not rule out hemolysis because in certain situations red blood cells do not decompose intravascularly. Whenever red blood cells are degraded due to a specific pathology, their altered morphology may help identify the underlying cause (Table 3).

**Table 2:** Classification of hemolytic anemias (modified from [5] and [10])

Intracorpuscular	***Abnormalities of red blood cell interior***	Hereditary
	Enzyme defects (e.g. deficiencies of glucose-6-phosphate dehydrogenase, pyruvate kinase, glucose-phosphate isomerase, 5’nucleotidase)	
	Hemoglobinopathies (e.g. sickle cell disease, thalassemia, unstable hemoglobin)	
	***Red blood cell membrane abnormalities***	
	Hereditary disorders (spherocytosis, elliptocytosis, pyropoikilocytosis, stomatocytosis)	
	Paroxysmal nocturnal hemoglobinuria	Acquired
Extracorpuscular	Spur cell anemia	
	***Extrinsic factors***	
	Hypersplenism	
	Antibodies (immune-mediated hemolysis)	
	Microangiopathic hemolysis (e.g. thrombotic thrombocytopenic purpura, hemolytic uremic syndrome, aortic stenosis, prosthetic valve leak)	
	Infection (e.g. *Bartonella, Babesia*, malaria, clostridial sepsis)	
	Intoxication (e.g. lead, copper, snake and spider bites)	
	Other reasons

**Table 3:** Red blood cell morphology in the diagnosis of hemolytic anemias (modified from [5] and [10])

Morphology	Cause	Syndromes
Spherocytes or microspherocytes	Loss of membrane	Hereditary spherocytosis, immunohemolytic anemia
Elliptocytes	Abnormality of spectrin leading to impaired assembly of the cytoskeleton	Hereditary elliptocytosis
	Deficiency in spectrin	Hereditary pyropoikilocytosis
Stomatocytes	Increased permeability to sodium and potassium	Hereditary stomatocytosis, “hydrocytosis”
Target cells and teardrop cells	Increased ratio of red blood cell surface area to volume	Thalassemia
Schistocytes, fragmentocytes	Traumatic disruption of cell membrane	Microangiopathy, intravascular prosthesis, microangiopathic hemolysis
Sickled cells	Polymerization of hemoglobin S	Sickle cell syndromes
Acanthocytes	Abnormal membrane lipids	Severe liver disease (spur cell anemia)
Agglutinated cells	Presence of IgM antibodies	Cold agglutinin disease
Heinz bodies	Precipitated hemoglobin	Unstable hemoglobin, oxidative stress
Blister or bite cells	Oxidant injury	Glucose-6-phosphate dehydrogenase deficiency

In view of an essentially unremarkable bone marrow report, laboratory data showing normal levels of leukocytes and platelets, and in the absence of splenomegaly, a hematologic malignancy can be ruled out in this case. A mildly to moderately hyperplastic bone marrow (erythroid hyperplasia) as seen in the bone marrow aspirate of the discussed patient usually suggests a response to anemia (e.g. blood loss or hemolytic anemia). The development of hyperplasia depends on the type, duration and severity of the anemia [11]. Hemosiderin pigment, as in this case, may be found in macrophages in the bone marrow associated with previous hemorrhage due to vascular injury or hemorrhagic diathesis. However, the iron content (hemosiderin) is also increased in anemia of chronic disease and can be used to differentiate this form of anemia from iron deficiency anemia, in which the marrow has a decreased iron content [11].

Paroxysmal nocturnal hemoglobinuria (PNH) is a hemolytic disorder caused by an intracorpuscular defect acquired at the stem cell level that manifests with hemolytic anemia, venous thrombosis and deficient hematopoiesis [12]. Red blood cells are normocytic normochromic unless iron deficiency has occurred from chronic iron loss in the urine (hemosiderinuria); leukopenia and thrombopenia are common and reflect impaired hematopoiesis [5]. In this rare condition with a prevalence of 4 per 100,000 [13], the absence of two glycosylphosphatidylinositol-anchored proteins, CD55 and CD59, leads to uncontrolled complement activation that accounts for hemolysis and the other PNH manifestations [14]. This activation also indirectly stimulates platelet aggregation and hypercoagulability, promoting venous thrombosis in affected patients, which primarily occurs in intraabdominal veins (hepatic, portal, mesenteric) and results in Budd-Chiari syndrome, congestive splenomegaly and abdominal pain [5]. The PNH should be suspected in patients with otherwise unexplained hemolytic anemia, especially with leukopenia and/or thrombopenia and with evidence of intravascular hemolysis (hemoglobinemia, hemoglobinuria, hemosiderinuria, elevated lactate dehydrogenase). Since these criteria are not met in the discussed patient, PNH can be excluded as a differential diagnosis. Moreover, hereditary red cell membrane disorders such as spherocytosis, elliptocytosis (including hereditary pyropoikilocytosis) and stomatocytosis (Table 3) can be ruled out as the underlying pathology of hemolysis in this case because there were no abnormalities found on complete blood cell count.

Hemolytic anemia is also a hallmark of hemoglobinopathies, which are genetic disorders affecting the structure, function or synthesis of hemoglobin. The two main groups are thalassemia syndromes (main types alpha‑ and beta-thalassemia) and structural hemoglobin variants (most frequently HbS sickle cell syndromes, HbE and HbC) [15]. Besides hemolytic anemia, erythrocytosis, cyanosis or vascular occlusive events may occur in different forms of this disease. Peripheral blood smear morphology and abnormalities of the complete blood cell count with erythrocyte indices and hemoglobin test (hemoglobin electrophoresis and/or chromatography) are pivotal for establishing the diagnosis [5, 15]. Since there were no abnormalities in the morphology of red blood cells in our patient, a hemoglobinopathy can be ruled out as the diagnosis. Other interior pathologies of red blood cells leading to hemolytic anemia and spherocytes on blood smear include enzyme defects (enzymopathies), particularly glucose-6-phosphate dehydrogenase deficiency [16]; however, in view of the constellation of laboratory findings and the clinical course, this cause seems unlikely in this case.

As the Coombs test was negative in our patient, immune-mediated hemolysis can be excluded. This leaves thrombotic microangiopathies, such as thrombotic thrombocytopenic purpura and hemolytic uremic syndrome as well as mechanical hemolysis as possible diagnoses; however, normal platelet counts, the absence of fragmentocytes on a blood smear and missing clinical features rule out the diagnosis of thrombotic microangiopathy.

Mechanically induced hemolysis is one of the potentially serious complications of prosthetic heart valves. Indeed, mild, compensated hemolysis associated with mechanical heart valves occurs frequently, while severe hemolysis is rare and usually reflects paravalvular leakage [17, 18]. Since the discussed patient did not have prosthetic heart valves, this consideration is not relevant in this case.

Pseudoacute abdomen and anemia may also be found in porphyrias, which are disorders of heme synthesis, each involving a defect (inherited or acquired) of specific pathway enzymes in the heme biosynthesis [19]. Porphyrias can be classified as hepatic or erythropoietic depending on the primary locus of overproduction or accumulation of the porphyrin precursors (delta-aminolevulinic acid, porphobilinogen) or porphyrin (uroporphyrin, coproporphyrin, protoporphyrin) [20]. Acute porphyrias are due to hepatic overproduction of the porphyrin precursors delta-aminolevulinic acid and porphobilinogen, and primarily manifest as neurological and neurovisceral symptoms (pseudoacute abdomen), as well as mental disturbances. In contrast, erythropoietic porphyrias are characterized by cutaneous photosensitivity due to overproduction of photosensitizing porphyrins by the liver and bone marrow [19]. The prevalence of some types of porphyria may be higher than generally assumed. The four acute hepatic porphyrias are acute intermittent porphyria, variegate porphyria, hereditary coproporphyria and delta-aminolevulinic acid dehydratase deficiency porphyria. Two of these, variegate porphyria and hereditary coproporphyria have both neurovisceral and cutaneous features [20]. Acute intermittent porphyria is the acute type most often encountered in clinical practice [21]. The prevalence of relevant mutations in the western population is about 1 per 2,000 persons [22, 23]; however, acute attacks occur in less than 10% of the at-risk population [24, 25]. Clinical expression of acute intermittent porphyria includes several days of fatigue and an inability to concentrate, followed by progressively worsening abdominal pain, nausea, vomiting and neurological symptoms in a previously healthy young person [19]. These attacks have a recurring character. Except for the gastrointestinal symptoms, this clinical course was not observed in the discussed patient and there were no such conditions documented in his history. Furthermore, urinalysis would not have been unremarkable if the patient had suffered from an attack of acute intermittent porphyria, which is characterized by urinary excretion of delta-aminolevulinic acid and porphobilinogen. Thus, urine turns purple when left at room air without protection from light for a few hours. Given these facts makes porphyria unlikely as a diagnosis in this case.

This leaves drug-induced or toxic causes on the list of differential diagnoses. About 125 different drugs have been identified to potentially cause hemolytic anemia with NSAIDs and anti-cancer drugs being the most prevalent [26, 27]. Neither medication was taken by our patient; however, about 6 weeks earlier he had been on a cephalosporin for 5 days. Indeed, cephalosporins are known to be potential inducers of clinically significant hemolytic anemia [27–30].

Drug-induced hemolytic anemia usually occurs within days or weeks of exposure to a drug and may be immune-mediated or nonimmune-mediated (oxidant injury). In case of immune-mediated hemolytic anemia, Coombs test (direct antiglobulin test) tends to be positive with IgG or complement activating drug dependent-antibodies of mainly IgM-type (immune complex type hemolytic anemia) attached to the surface of red blood cells [27, 29]. The molecular mechanism of cephalosporin-induced hemolytic anemia is still poorly understood, but an immune-mediated process is suggested, even though varying degrees of direct antiglobulin test activity have been reported in affected patients [27]. In view of the constellation of a negative Coombs test and a period of 6 weeks between termination of cephalosporin treatment and onset of symptoms, drug-induced hemolytic anemia does not seem to be the underlying cause of the patient’s complaints.

Thus, toxic agents, which have the potential to cause abdominal pain, gastrointestinal complaints and anemia should be considered. In this context, heavy metal poisoning has to be discussed. Heavy metals such as arsenic and lead pose a significant threat through occupational or environmental exposure. They are inhaled primarily as dusts and fumes (tiny particles generated by combustion) or vapors. When metals are ingested in contaminated food or drinks or by hand to mouth activity, the gastrointestinal absorption varies greatly with the specific chemical form of the metal and the nutritional status of a person. Once absorbed, heavy metals are mainly transported in the blood, with the precise kinetics dependent on diffusibility, protein binding, rates of biotransformation, availability of intracellular ligands and other factors [5]. Arsenic is one of the most toxic metals derived from nature. Globally, the major cause of human intoxication is the consumption of drinking water contaminated from natural geological sources, with Bangladesh and West Bengal (India) being the most affected areas [31]. Arsenic is used in the smelting and microelectronics industry (e.g. light-emitting diodes, lasers) and is contained in wood preservatives, pesticides, herbicides, fungicides and coal (and released during incineration). This metal occurs in two oxidation states, arsenite (trivalent form) and arsenate (pentavalent), with arsenite being 60 times more toxic than arsenate [31]. In the human body, arsenic inactivates about 200 different enzymes, most of them involved in cellular energy pathways and DNA replication and repair, and substitutes phosphate in high energy compounds such as adenosine triphosphate. Moreover, it exerts its toxicity by causing increased oxidative stress and by binding thiol or sulfhydryl groups in tissue proteins of the liver, lungs, kidneys, spleen, gastrointestinal mucosa and keratin-rich tissues [31, 32]. Exposure occurs from ingestion, inhalation and absorption by the skin. Clinical features of acute intoxication include nausea, vomiting, bloody diarrhea and colicky abdominal pain caused by necrosis of intestinal mucosa and hemolysis [5]. Thereby, abdominal pain may mimic acute abdomen [33]. Profuse watery diarrhea, which is attributable to increased permeability of the blood vessels, is a dominant feature. The voluminous watery stools are also called “choleroid diarrhea” or “bloody rice water diarrhea” and cause severe fluid loss and dehydration [34]. Furthermore, signs and symptoms may include excessive salivation, pulmonary edema, acute tubular necrosis, delayed cardiomyopathy but also neurological symptoms, such as delirium or seizures. Chronic exposure causes symptoms such as sensory and motor polyneuritis, and skin changes such as hyperkeratosis, hyperpigmentation, exfoliative dermatitis and Mee’s lines (transverse white striae of the fingernails) [5]. Intoxication with arsenic may further lead to hepatomegaly, hepatitis and alteration in hepatic architecture (steatosis and noncirrhotic portal fibrosis), resulting in portal hypertension without cirrhosis [34, 35]. Although laboratory data of our patient showed elevated transaminases and cholestasis, these alterations were too mild and did not fulfill the criteria of toxic or drug-induced liver injury [36]. Furthermore, imaging studies did not reveal hepatomegaly or pathological changes of liver structure such as steatosis or fibrosis, making the diagnosis of arsenic poisoning unlikely in this case.

Focusing on the potential exposure to heavy metals, one should keep in mind that the patient had spent several days working on the demolition of an old bridge where he was exposed to considerable dust 6 weeks prior to admission. In former days, red lead (Pb_3_O_4_) was frequently used for anti-rust paints (corrosion protection of steel constructions such as bridges, ships and locomotives), which are nowadays banned from the market because of their toxicity [37]. Since the bridge which had been demolished by the patient was old (built before 1975), it may have been coated with red lead and the patient may have been exposed to significant amounts of lead in the course of his work. Lead enters the body by absorption through ingestion, inhalation or via dermal absorption [5]. Symptoms of intoxication include, among others, pseudoacute abdomen, anemia caused by impaired hematopoiesis, and arthralgia as found in the discussed patient. Anemia associated with lead poisoning can be normocytic or microcytic; it is attributable to several mechanisms including (1) impaired heme synthesis (interference with two of the enzymes involved in heme synthesis: delta-aminolevulinic acid dehydratase and ferrochelatase), (2) shortened survival of red blood cells (hemolysis due to increased osmotic fragility and changes of the red blood cell shape) and (3) impaired renal production of erythropoietin [38]. Inhibition of the insertion of iron in the porphyrin ring results in the formation of free protoporphyrins or zinc protoporphyrin if zinc is substituted [39]. Physical examination may further reveal a Burton’s line (“lead line”) at the gingiva-tooth border which develops when lead reacts with oral bacterial metabolites in patients with chronic exposure [40]. In view of the entire constellation of findings in this case, lead poisoning seems to be the most likely diagnosis. This should be confirmed by analysis of lead in whole blood and urine.

## Dr. F. Keil’s diagnosis

Lead poisoning

## Discussion of case

### Dr. K. Hegenbarth:

The history of working on the demolition of an old bridge with exposure to considerable dust followed by persistent stabbing abdominal pain, vomiting and weight loss strongly suggested lead poisoning in this patient. To confirm this, the concentration of lead in whole blood was analyzed; at 92 µg/dL (normal: 0–45 µg/dL), the level was clearly elevated as was the excretion of lead in 24h-urine (244 µg/L, normal: < 40 µg/L). For the management of lead poisoning, chelating agents such as dimercaprol (2,3-dimercapto-1-propanol, British Anti-Lewisite (BAL)), calcium disodium ethylenediaminetetraacetic acid (CaNa_2_-EDTA), succimer (meso-2,3-dimercaptosuccinic acid, DMSA) and D‑penicillamine are available [41, 42]. In the presented case, the patient was treated with 2,3-dimercapto-1-propanesulphonic acid (DMPS, Dimaval^®^ (Heyl, Berlin, Germany)). He received an initial dose of 1250 mg i.v., then 120 mg orally for 6 days, followed by 400 mg orally for 3 days. Two days after the initiation of treatment, the patient was discharged free of complaints.

### Dr. E. Beubler:

The history in this case immediately suggests lead poisoning from red lead (“Mennige”), which is an orange-brown anti-rust paint that was only banned in the 1980s. This paint originally gave the Golden Gate Bridge in San Francisco its distinctive color. Lead is one of the most poisonous metals with a maximum permissible concentration at the workplace of 0.1 mg/m^3^ [43]. In the past, lead poisoning was frequently iatrogenic owing to lead acetate (lead[II]acetate, liquor plumbi subacetici), lead ointments, lead plasters, lead nipple caps for tumors and aqua plumbi for eye compression [44]. The natural emission is 18.6 × 10^3^ tons/year, anthropogenic emission is 483 × 10^3^ tons/year, with 70% from motor vehicle exhaust. The rest is from iron and steel industry, and coal combustion [45]. Lead pipes in the home can also be a source of poisoning, especially with pipes that are not yet sulfated inside, and with soft carbonated water. Humic acids in soil can change water quality and increase the lead load. The World Health Organization (WHO) maximum allowance for lead in drinking water is 0.05 mg/L [46]. In Austria (since 2013) the maximum allowance for lead in drinking water is 0.01 mg/L [47, 48].

Acute lead poisoning is very rare. It causes severe colicky pain and a lead encephalopathy that, left untreated, is fatal in 30% of cases. With chronic poisoning, encephalopathy can manifest as a lead crisis when there is renewed uptake or mobilization of lead. Lead poisoning damages the blood and nervous system, skin, mucous membranes, smooth muscles and the skeleton. Blood work shows anemia with basophilic stippling in the erythrocytes; in the bone marrow, there is increased erythropoiesis, also with basophilic stippling. There is damage to the motor nerves, especially the radial nerve, with wrist drop, saturnine encephalopathy with fatigue, insomnia, muscle tremors including epileptiform seizures, and intolerance to alcohol. The skin shows a yellow-grayish pallor, a lead hue of the skin due to anemia and spastic contractions of the blood vessels in the skin. Moreover, lead reacts with oral bacterial metabolites (H_2_S) which leads to formation of lead sulfide. In the gastrointestinal tract, smooth muscle spasm causes colicky pain. Contraction of the capillaries and arterioles causes lead nephritis and contracted kidneys. In children, there are “lead lines”, i.e. lead deposits in the growth zones of the bones. The course is chronic and the onset often gradual. Lead in bone has a half-life of 30 years, so even with leaching, chronic poisoning cannot be cured since the bones continuously release lead [49]. Leaching agents include Dimaval^®^ (Heyl, Berlin, Germany), CaNa_2_-EDTA or D‑penicillamine [50].

### Dr. G. J. Krejs:

Today, lead poisoning is very rare in medical and neurological patients. In the 35-year history of clinical-pathological conferences at the Medical University of Graz this is the only such case. Although it is uncommon in Austria, in the USA, X‑rays often show incidental projectiles in the body. In Texas, shotgun injuries often leave buckshot in the body, but lead does not tend to be liberated into the body and poisoning usually does not occur; however, if the projectile is in an acidic environment, e.g. near sites of chronic arthritis, it can be another matter. At Southwestern Medical School in Dallas, we once had an African-American woman with severe spondyloarthritis. Her son had taken a potshot at her and for nearly a year a projectile was lodged near an intervertebral disc close to such a spondyloarthritic site. The patient was admitted with severe neurological symptoms and anemia and died before the result of her serum lead level of 500 µg/dL became available. The charge against her son was changed from aggravated assault to manslaughter. A similar case with lead poisoning after a gunshot to the right elbow 6 years earlier, was reported by Shen [51]; the patient presented with back pain and abdominal pain, nausea and constipation for several weeks. Laboratory data revealed normocytic anemia with basophilic stippling of the erythrocytes. Lead poisoning has also been reported in Alaska Natives who ingested lead shot embedded in the meat of hunted waterfowl. Although most of the metal is passed in the feces, buckshot may accumulate in the appendix and may lead to intoxication with lead over time [52, 53].

Lead poisoning has already been described in antiquity [54] and has been with mankind ever since. While some attribute its first mentioning to Hippocrates around 500 BC, the first description that fits modern understanding of the disease, i.e. lead colic (abdominal pain), peripheral neuropathy (wrist drop), encephalitis (mental confusion) and anemia (pallor), is found in a poem by Nikander of Colophon in 200 BC [55]. In 1767, George Baker identified lead poisoning as the underlying cause of the Devonshire colic, also known as dry gripes (abdominal pain without diarrhea), due to the consumption of West Indian rum contaminated with lead. For this essential revelation he was aided by Benjamin Franklin [56]. About one century later, Sir Alfred Baring Garrold identified saturnine gout as an additional symptom of lead poisoning when he recognized that many of his gout patients were lead workers [57]. Indeed, uric acid may contribute to the development of hypertension and renal failure in lead poisoning [58, 59]. Baker’s classical description remained the clinical standard for recognition of acute lead poisoning until the twentieth century when blood lead measurement became available [56].

Inorganic lead is the most extensively studied toxic agent and its history is closely linked to safety, toxicity and politics [60]. The annual world mine production of lead is about 4.5 million tons, with Asia and the USA being the most important producers; the world’s lead processing is significantly higher (about 12 million tons, including recycled lead) [61]. Today, the predominant use (80%) of lead is in batteries, mainly for vehicles, but also for electricity back-up systems and industrial batteries. Furthermore, lead is used for rolled and extruded products (6%), as pigment (5%), and in ammunition (3%) [62], as cable sheathing, solders, alloys (brass and bronze), weights, crystal and as a stabilizer in polyvinyl chloride [60].

The history of pollution is very long. Around 3500 BC, a method for extracting silver from lead ores appeared; this subsequently led to an increasing release of the by-product lead into the environment, peaking during the age of the Roman Empire [63, 64]. In this period, there was widespread use of lead technology (e.g. in aqueducts) and lead acetate was used as sweetener in wine. In the nineteenth century, lead-containing tin was widely used in household utilities. Lead also had widespread use in paint, both for corrosion protection of steel constructions such as bridges, ships and locomotives, and in houses. In some countries, white lead (lead carbonate hydroxide) was even commonly used for interior painting, furniture and toys, making up to 40% of the final dried solid [37]. Moreover, it has also been frequently used in water piping and in the fitting of water pipes [60]; however, lead content of tap water may vary considerably from < 5 µg/L to 330 µg/L [65]. Indeed, lead level of tap water strongly depends on how long water has been sitting in the pipe. Since the content is often highest in the first flush, flushing water through the pipes before drinking can significantly lower lead levels [65]. During the twentieth century, enormous amounts of the organolead compounds tetraethyl lead and tetramethyl lead (about 1 g/L) were used as an anti-knock agent in gasoline. At combustion in the engine, organic lead is transformed into inorganic lead oxide and emitted almost entirely, causing significant lead exposure in people living in areas with heavy traffic [60]. Annual global lead emission into the environment was about 400,000 metric tons during the 1960s and 1980s, but has significantly decreased after the phaseout of lead in gasoline [54, 66].

Today, occupational exposure in pottery painting and glazing [67] but also some contaminated herbal remedies [68–73] can still be a source of lead exposure. Moreover, cigarette smoking exposes to inhalation of lead; however, while only 2% of contained lead will be inhaled by the active smoker, most of the lead is released with the tobacco smoke. Consequently, there is an association between children’s lead exposure and environmental tobacco smoke [74, 75]. Regarding ammunition, lead exposure occurs both through the ingestion of game meat [76, 77] and occasionally from lead bullets retained in the human body that may release significant amounts of lead as described above.

In Europe, the mean dietary exposure among 19 countries is about 0.51 µg/kg body weight per day, corresponding to about 35 µg/day for a 70-kg adult [78]. Although multiple food groups contribute to dietary lead exposure, cereals, vegetables (potatoes, leafy vegetables) and tap water are at the top of the list [78].

As described in the present case, abdominal pain (pseudoacute abdomen) is a hallmark and sometimes the only clinical symptom in lead poisoning. It is suggested that colicky pain occurs when blood levels exceed 80 µg/dL, whereas levels higher than 60 µg/dL result in milder nonspecific gastrointestinal discomfort and constipation [39]. While intermittent abdominal cramps localized in the hypogastrium (sometimes in the epigastrium), protracted constipation, tenesm, indigestion, vomiting and loss of appetite are common, diarrhea is only observed occasionally. Indeed, the clinical picture may be mistaken for intestinal obstruction or appendicitis [60]. Mechanistically, it is suggested that lead as well as delta-aminolevulinic acid, the level of which is increased as a consequence of porphyrinopathy in lead poisoning [79], have negative effects on intestinal motility [80], the enteric nervous system and smooth muscles [79, 81]. Lead interferes with sodium transporter channels of the intestine [79], but it is also suggested that abdominal pain is due to interaction with calcium in the smooth muscle cells. Impaired calcium homeostasis caused by lead exposure will result in apoptosis of neuronal cells [82]. In addition, lead disrupts the normal fluid homeostasis in neurons which results in high cellular pressure and consequent segmental demyelination in neurons [83]. Due to the involvement of calcium in the development of abdominal pain, intravenous administration of calcium will give temporary relief [60].

## Final diagnosis

Lead poisoning
